# Multi-model GWAS reveals key loci for leaf angle in maize inbred lines from southeast China

**DOI:** 10.1186/s12870-026-09162-w

**Published:** 2026-06-03

**Authors:** Keyu Li, Jia Xie, Jinghan Cao, Changjin Wang, Wangfei He, Renjie She, Ruzhen Bao, Yuchen Chai, Haoze Yu, Yu Han, Weidong Huang, Xinxin Cheng, Mingliang Cui, Lei Chen, Haibing Yu

**Affiliations:** 1 College of Agriculture, Maize Germplasm Repository of Anhui Province, Anhui Science and Technology University, Chuzhou, 233100 China; 2 Suixi County Agricultural Research Experimental Station, Huaibei, 235000 China; 3 SDIC Fengle Seed Co., Ltd, Hefei, 230000 China

**Keywords:** Maize, Inbred germplasm, Genome-wide association studies, Leaf angle, Candidate genes, KASP markers

## Abstract

**Background:**

Maize is a vital grain and forage crop with continuously increasing yield demands, making the improvement of plant architecture for high-density planting particularly significant. Among architectural traits, leaf angle (LA) is a key factor influencing light interception and canopy architecture. To dissect the genetic basis of LA in maize, a genome-wide association study was conducted.

**Results:**

A panel of 212 maize inbred lines from breeding programs in Southeast China was genotyped using the Maize6H-60 K SNP array. Employing five GWAS models (GLM, MLM, SUPER, FarmCPU, and BLINK), we identified 14 significant SNPs (*p* < 8.55 × 10⁻⁷) and 342 candidate genes within the 200 kb flanking regions, 134 of which were functionally annotated. Multi-model cross-validation revealed six stably associated SNPs, including four novel loci (AX-107936049, AX-108102817, AX-107953621, and AX-108061477). AX-86,305,869 was located within 2 Mb of a previously reported significant SNP, while AX-107,994,145 fell within a known QTL interval. Within these six stable loci, 11 high-confidence candidate genes are putatively associated with LA variation, potentially acting via hormone signaling, leaf morphogenesis, and midvein development. Notably, *GRMZM2G036905* (homologous to *Arabidopsis ELF1*) may play a role in regulating upright leaf architecture, and *GRMZM2G003565* may contribute to leaf rolling as previously suggested. Based on these stable SNPs, KASP markers were developed and validated in 52 varieties/lines (including Zhengdan 958, MY73, and Anke 985) and 20 inbred lines, confirming the association between these SNPs and LA variation.

**Conclusion:**

Our study identified six stable SNPs and 11 candidate genes for maize LA through multi-model GWAS in 212 inbred lines. Four novel loci were discovered, and KASP markers were developed for three stable SNPs, providing genetic resources and tools for breeding maize varieties tolerant to high planting density.

**Supplementary Information:**

The online version contains supplementary material available at 10.1186/s12870-026-09162-w.

## Introduction

Maize (*Zea mays* L.) is a major global food crop with extensive applications in human consumption, animal feed, and industrial processing [1]. Its yield level is crucial for ensuring food security and supporting economic development. While global maize production has maintained steady growth [2], demand is projected to rise further due to population increase and economic development. By 2033, global grain consumption is projected to reach 3.2 billion metric tons, with maize accounting for the largest share [3]. To address future supply pressures, genetic improvement of plant architecture to enhance yield potential has become a key direction in maize breeding [4–6].

Leaf angle (LA), defined as the angle between the leaf midrib and the stem [7, 8], is a key determinant of ideal plant architecture in maize [6, 9]. LA directly influences light interception efficiency and tolerance to high planting density, thereby affecting overall canopy light utilization and ultimately grain yield [10–12]. Elucidating the genetic basis of LA is therefore essential for optimizing plant architecture and improving yield in maize.

Recent advances in molecular biotechnology and high-throughput sequencing have greatly promoted the application of genome-wide association studies (GWAS) in dissecting the genetic architecture of agronomic traits in crops such as cotton [13], maize [14], rapeseed [15], rice [16], and wheat [17]. This approach detects statistical associations between phenotypic variation and genome-wide genetic markers across diverse germplasm panels, enabling the identification of loci underlying complex traits [18]. Accumulating evidence from GWAS has advanced our understanding of the genetic basis underlying maize LA. For example, Lu et al. performed GWAS using 80 key maize inbred lines across two years, identifying 22 significant SNPs and candidate genes, five of which were validated via qRT-PCR [19]. In another study, Duan et al. conducted GWAS for LA using 492 inbred lines evaluated in five environments, detecting 85 significant SNPs and revealing seven meta-QTLs [20]. Similarly, Peng et al. analyzed LA phenotypes collected over three years in 285 inbred lines, identifying 96 significant SNPs and predicting seven candidate genes [21].

Current evidence indicates that genes regulating LA function mainly through pathways controlling auricle and ligule development (e.g., *LG1*, *LG2*, *ZmBEH1*) [22–24], establishment of leaf polarity (e.g., *MWP1*, *ZmHB53*) [25, 26], phytohormone signal transduction (e.g., *LGN*, *Brachytic2*, *ZmILI1*, *ZmACS7*, *UPA1*, *UPA2*) [27–30], and regulation of mechanical tissue strength in the midrib (e.g., *ZmDRL1*, *ZmDRL2*) [31]. A recent study employing map‑based cloning identified two additional genes on chromosome 4 that regulate maize LA, encoding a 3‑hydroxyacyl‑CoA dehydrogenase (*AIM1*) and a RING‑type E3 ubiquitin ligase (*SINAT4*), respectively [32]. Although a number of regulatory genes have been characterized, they likely do not represent the full genetic architecture of this trait. Therefore, further systematic mining of associated loci and candidate genes is necessary to elucidate the molecular mechanisms underlying LA variation.

Despite an increasing number of studies reporting LA-associated QTL and genes, only a limited subset has been effectively applied in maize breeding. A primary reason is that, following long-term selection during crop improvement, favorable QTLs and alleles may have reached high frequency, near fixation, or fixation within elite germplasm used in specific breeding programs. As a consequence, loci identified from diverse germplasm panels may not maintain sufficient polymorphism within these breeding populations to support further genetic improvement [33]. Moreover, due to differences in genetic materials, environmental conditions, and analytical methods across studies, the significant SNPs detected often vary [34]. When the target breeding environment differs substantially from the original conditions, the results tend to show limited reproducibility and reduced breeding effectiveness. Therefore, further in-depth investigation into the genetic mechanisms of LA in maize inbred lines derived from breeding programs in southeastern China is warranted. In this study, an association panel consisting of 212 maize inbred lines was used, together with LA phenotypic data collected over two consecutive years. Five models (GLM, MLM, SUPER, FarmCPU, and BLINK) were employed to identify QTL regulating LA and to screen candidate genes. Furthermore, KASP markers were developed based on significant SNP, providing a theoretical basis and practical tools for fine mapping, gene cloning, and molecular breeding of ideal plant architecture in maize.

## Materials and methods

### Plant materials and field experiment design

The 212 maize inbred lines used in this study were obtained from the Maize Breeding Engineering Technology Research Institute of Anhui Province, China [35]. Field trials were conducted in Fengyang County, Anhui Province (32°N, 117°E) during the 2023 (23FY) and 2024 (24FY) growing seasons, constituting two distinct environments. A single-row plot design was adopted with the following specifications: row length of 5.00 m, row spacing of 0.60 m, and plant spacing within rows of 0.33 m. The experiment was arranged with two replications. All field management practices followed standard agronomic protocols for maize cultivation.

### Phenotypic measurements

In this study, LA phenotyping was conducted at the early milk stage, when maize leaves remained green and had not yet senesced. Five representative plants per inbred line were randomly selected from each of the two replicates, and the measured leaves were ensured to be intact and free from severe senescence. The LA was measured as the angle (°) between the leaf midrib and the stem for three consecutive leaves above the ear [7, 8]. The average value of these measurements was considered the phenotypic value for each plant. Descriptive statistics—including mean, variance, standard deviation, coefficient of variation, range, skewness, and kurtosis—were computed using Microsoft Excel 2021 and IBM SPSS Statistics 23.0. Pearson correlation analysis and normality tests were also performed. For analysis across the two-year environments, best linear unbiased estimates (BLUEs) of LA across both years were estimated using the lmer function from the *lme4* package in R [36]. Genotype was treated as a fixed effect, while replicate and year were included as random effects in the model. Broad-sense heritability was estimated using the following formula:

$$\:{H}^{2}=\frac{{\sigma\:}_{g}^{2}}{\left({\sigma\:}_{g}^{2}+\frac{{\sigma\:}_{ge}^{2}}{n}+\frac{{\sigma\:}_{e}^{2}}{nr}\right)}$$

In the formula, $\:{\sigma\:}_{g}^{2}\:$ denotes genetic variation, $\:{\sigma\:}_{ge}^{2}$ represents the interaction between genotype and environment, $\:{\sigma\:}_{e}^{2}$ indicates residual error, n refers to the number of environments, and r signifies the number of repetitions within each environment [37].

### Genotyping

Genotyping was performed using the Maize6H-60 K SNP array developed by the Maize Research Center, Beijing Academy of Agriculture and Forestry Sciences [38]. SNP loci with a minor allele frequency (MAF) below 5% and a missing rate exceeding 20% were excluded. A total of 58,455 high-quality SNP markers were retained for subsequent analyses. PopLDdecay 3.40 was used to evaluate genetic relationships among individuals and estimate linkage disequilibrium (LD) based on the coefficient (r²), from which an LD decay plot was generated [35].

### Genome-wide association study

Genome-wide association analysis of maize LA was conducted using five models implemented in the R package GAPIT version 3.4 [39, 40]: GLM (General Linear Model), MLM (Mixed Linear Model), SUPER (Settlement of MLM Under Progressively Exclusive Relationship), FarmCPU (Fixed and Random Model Circulating Probability Unification), and BLINK (Bayesian-information and Linkage-disequilibrium Iteratively Nested Keyway).

Based on our previous work, the first three principal components (PCs) were incorporated as covariates in all models to control for population structure. GLM is computationally efficient but tends to produce inflated false positives due to the absence of kinship correction. The kinship matrix (K) was calculated using PLINK 1.9 and incorporated into the MLM model, which accounts for relatedness and reduces false positives, albeit often at the cost of statistical power [41]. Notably, the SUPER model does not rely on a genome-wide kinship matrix constructed from all markers; instead, it uses an optimized relationship matrix derived from a subset of associated markers [42]. In multi-locus approaches, FarmCPU iteratively fits fixed- and random-effect models, using pseudo quantitative trait nucleotides (QTNs) to control background confounding [43]. BLINK further replaces the random‑effect model with a fixed‑effect model and selects markers based on Bayesian information criteria and linkage disequilibrium [39, 40].

To control for false positives, the Bonferroni correction was applied, resulting in a significance threshold of –log₁₀(p) > 6.07. The validity of the association results was assessed using Q-Q plots and p-value distributions. To evaluate potential inflation of the GWAS test statistics, the genomic inflation factor lambda (λ) was calculated using R according to the following formula: λ = median(qchisq(1 − p, 1))/qchisq(0.5, 1), where p represents the vector of P-values from the GWAS results. A λ value close to 1 indicates no obvious inflation of the test statistics [44]. Candidate genes were predicted based on the linkage disequilibrium (LD) decay characteristics of the population, with gene annotations retrieved from the MaizeGDB database (B73 RefGen_v3 served as the reference genome for SNP design). The LD decay distance was estimated as 200 kb (r² = 0.15) [35].

### Functional annotation of candidate genes

For significant SNP loci identified through association analysis, candidate genes were defined as those located within a 200 kb genomic region (upstream and downstream) of each marker, based on LD structure characterization [35]. Functional prediction and annotation of candidate genes were performed using the following biological databases: MaizeGDB (https://maizegdb.org/gbrowse/maize_v3/), NCBI (https://www.ncbi.nlm.nih.gov/), TAIR (https://www.arabidopsis.org/), and UniProt (https://www.uniprot.org/).

Gene Ontology (GO) and Kyoto Encyclopedia of Genes and Genomes (KEGG) enrichment analyses were conducted using the agriGO tool (https://systemsbiology.cau.edu.cn/agriGOv2/), the KEGG database (https://www.kegg.jp/), and the GeneCloud platform (https://www.genescloud.cn/).

RNA-seq data were obtained from the Maize Gene Function and Expression Database (MaizeGDB). A heatmap of candidate gene expression was generated using the R programming language. Gene expression was considered detectable at a threshold of FPKM ≥ 1, and expression levels were normalized using the formula log₂(FPKM + 1) [45]. Protein–protein interaction (PPI) networks were constructed using the STRING database (https://string-db.org/) and visualized with Cytoscape software (v3.10.3).

### Characterization of superior allelic variants at significant loci and KASP markers development

Based on the association analysis results, allelic effect analysis was performed for the stable SNP loci identified. Group differences were assessed using t-tests, and corresponding box plots were generated with GraphPad Prism 8 and Origin 2021. KASP primers (Table S6) were designed using PrimerPicker version 6. Each 10 µL KASP reaction contained 1 µL of genomic DNA, 5 µL of 2× KASP Master Mix (Vazyme, Nanjing, Q113-03), 3.6 µL of deionized water, 0.1 µL each of the allele-specific primers SNP-F1 and SNP-F2, and 0.2 µL of the common reverse primer SNP-R. The amplification protocol consisted of an initial denaturation at 95 °C for 5 min; followed by 40 cycles of 95 °C for 10 s and 60 °C for 30 s; and a final extension at 72 °C for 2 min. The KASP assays were performed on a real-time PCR instrument. Fluorescence signals were monitored during the annealing/extension step of each PCR cycle. After amplification, genotype calling was automatically performed by the instrument’s software based on the kinetic fluorescence data.

## Results

### Phenotypic analysis of LA

**Fig. 1:**
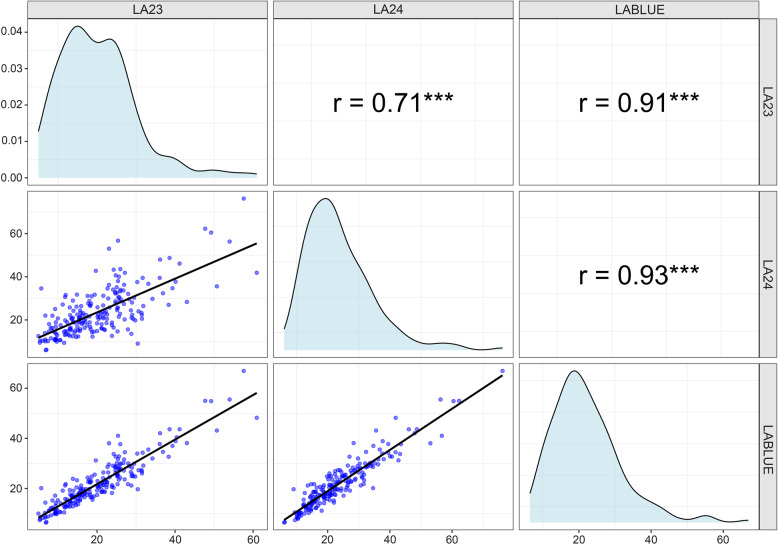
Correlation analyses between leaf angle phenotypes of the 212 maize inbred lines. *** indicates *P* < 0.001

Phenotypic analysis revealed that LA varied between 4.98° and 76.23° across environments (Table 1; Table S1). The coefficients of variation were 49.03% in the 23FY environment and 46.19% in 24FY (Table 1), indicating substantial phenotypic variation among the 212 maize inbred lines. The skewness and kurtosis of LA distribution were 1.18 and 2.19 in 23FY, and 1.44 and 3.25 in 24FY, respectively (Table 1). Broad-sense heritability (H²) of LA reached 82.7% (Table 1), indicating that genetic factors predominantly control this trait. Correlation analysis showed a highly significant positive relationship (*r* = 0.71, *P* < 0.001) between LA measurements in 2023 (LA23) and 2024 (LA24) (Fig. 1). Moreover, LA values in both environments were strongly correlated with their best linear unbiased estimates (BLUEs; *r* = 0.91 for 23FY and *r* = 0.93 for 24FY, *P* < 0.001; Fig. 1).

**Table 1:** Phenotypic variation of leaf angle (LA) in 212 inbred lines of maize

Traits	environment	Mean ± SEM	Range	Skewness	Kurtosis	CV (%)	H^2^(%)	Variance
LA	23FY	20.36 ± 9.98	4.98–60.93	1.18	2.19	49.03	82.7	99.59
	24FY	23.67 ± 10.93	6.00–76.23	1.44	3.25	46.19		119.50

### Genome-wide association analysis

Genome-wide association analysis of LA was conducted using five models in the GAPIT package: GLM, MLM, SUPER, FarmCPU, and BLINK. A significance threshold of *p* < 8.55 × 10⁻⁷ (–log₁₀(p) > 6.07) was applied. The Q-Q plots indicated varying degrees of deviation among the models. The genomic inflation factors (λ) for the five models were as follows: GLM (2.0151), MLM (1.0176), SUPER (1.8006), FarmCPU (1.0472), and BLINK (1.1589). the genomic inflation factors for MLM, FarmCPU, and BLINK were within a marginally acceptable range, demonstrating improved control of false positives and population structure.

**Fig. 2:**
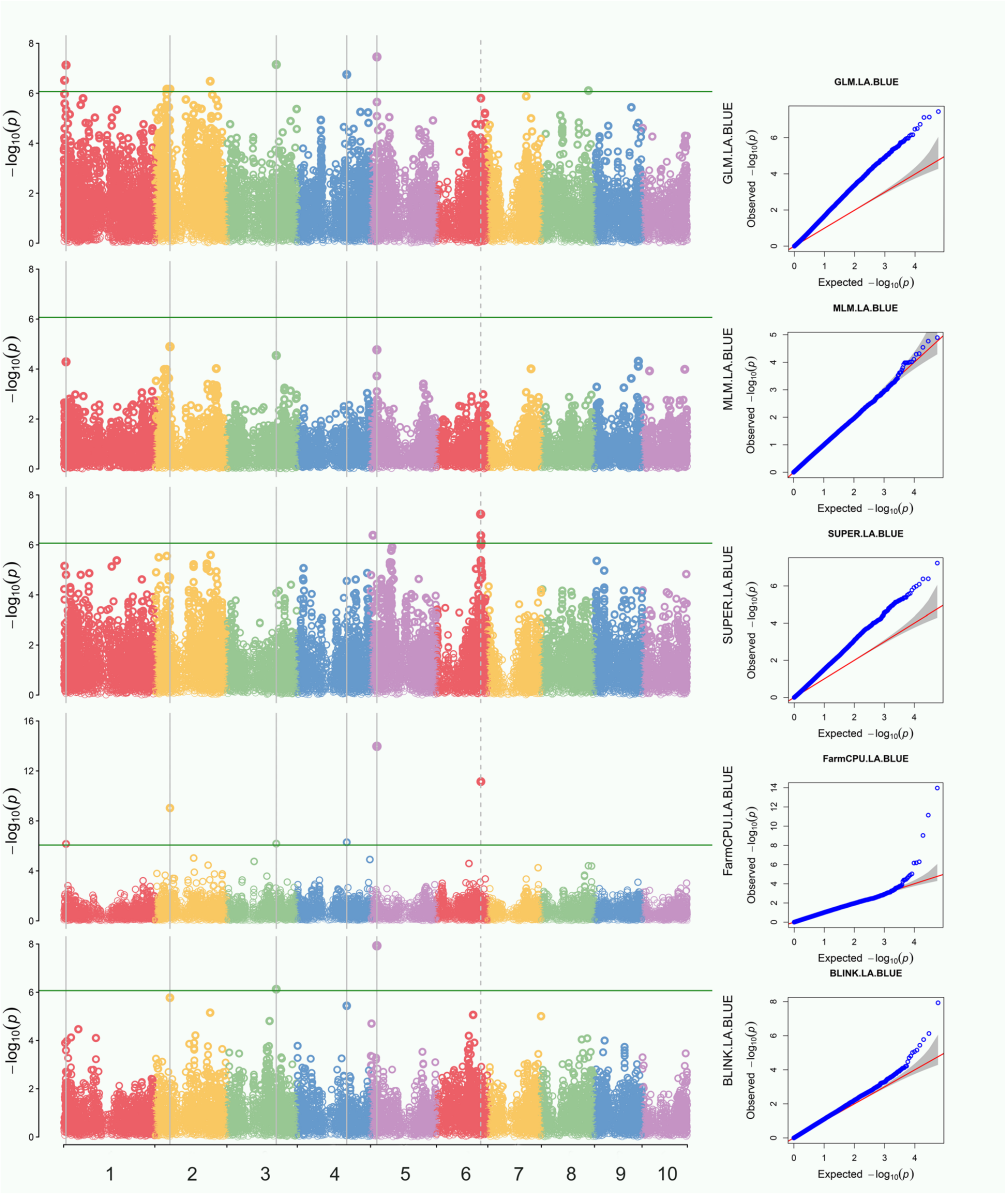
Manhattan plots (left) and quantile–quantile (QQ) plots (right) for leaf angle. The Manhattan plots display SNP associations across chromosomes (color-coded by chromosome). The horizontal green line indicates the genome-wide significance threshold (− log₁₀(P) = 6.07, corresponding to *P* < 8.55 × 10⁻⁷). The QQ plots show the observed versus expected − log₁₀(P) distributions; the red line represents the expected line under the null hypothesis

Fourteen significant SNPs associated with LA were identified by four models (GLM, SUPER, FarmCPU, and BLINK), distributed across seven chromosomes (excluding 7, 9, and 10) (Fig. 2, Table S2). Among the detected SNPs, three were identified by two models and two by three models; These loci were considered stable due to their reproducible detection across models, and the well-behaved Q-Q plots observed in at least one model, without evident inflation or excessive conservativeness. Notably, the locus AX-86,305,869 on chromosome 3 was consistently identified by GLM, FarmCPU, and BLINK (Fig. 2; Table 2), with P-values of 7.08 × 10⁻⁸, 6.61 × 10⁻⁷, and 7.47 × 10⁻⁷, respectively, and exhibited relatively high phenotypic variance explained (PVE) across all three models (Table 2). Another key SNP, AX-107,953,621 on chromosome 5, was also detected by the same three models, with P-values of 3.50 × 10⁻⁸, 1.07 × 10⁻¹⁴, and 1.18 × 10⁻⁸. Additionally, three stable SNP—AX-107,936,049 (chromosome 1), AX-108,102,817 (chromosome 2), and AX-107,994,145 (chromosome 4) were co-detected by GLM and FarmCPU (Table 2; Table S2). Two SNP loci on chromosome 6, AX-108,061,477 (detected by the SUPER model) and AX-108,014,790 (detected by the FarmCPU model), were located within ± 200 kb of each other (Table 2; Table S2). Given their colocalization within the ± 200 kb interval, AX-108,061,477 was also designated as a stable SNP locus in subsequent analyses.

**Table 2:** Identified stable SNP loci and predicted candidate genes

Trait	Marker name	Chr.	Position(bp)	Model		*P*-value	PVE(%)	Candidate gene	Gene Annotation
LA	AX-107,936,049	1	7652821		GLM	7.43E-08	2.19	*GRMZM2G059428*	NAC-transcription factor 53
					FarmCPU	6.95E-07	1.65E-07		
	AX-108,102,817	2	49740954		GLM	6.76E-07	1.57	*GRMZM2G003565*	Regulator of chromosome condensation2
					FarmCPU	9.31E-10	1.82	*AC186329.3_FG009*	Tetratricopeptide repeat (TPR)-like superfamily protein
								*GRMZM2G436187*	Transducin family protein/WD-40 repeat family protein
	AX-86,305,869	3	162842787		BLINK	7.47E-07	49.77	*GRMZM2G133173*	Ribophorin II (RPN2) family protein
					FarmCPU	6.61E-07	24.93	*GRMZM2G133398*	VP1, dormancy regulatory protein
					GLM	7.08E-08	12.86		
	AX-107,994,145	4	163227890		FarmCPU	5.14E-07	4.84	*GRMZM2G175983*	Auxin efflux carrier family protein
					GLM	1.78E-07	6.28	*GRMZM2G036905*	Component of the RNA polymerase II elongation complex
								*GRMZM2G092085*	Transducin/WD40 repeat-like superfamily protein
	AX-107,953,621	5	20828657		BLINK	1.18E-08	13.37	*GRMZM2G011156*	Cytochrome P450 36
					FarmCPU	1.07E-14	0.00		
					GLM	3.50E-08	7.35		
	AX-108,061,477/AX-108,014,790	6	146129498/146225884		SUPER/FarmCPU	4.21 E-07/ 7.14 E-12	4.71/ 0.00	*GRMZM2G173630*	Encodes a gibberellin (GA) receptor, has GA-binding activity, showing higher affinity to GA4.

*Chr.* Chromosome, *PVE* Phenotypic variance explained

### Candidate gene analysis

**Fig. 3:**
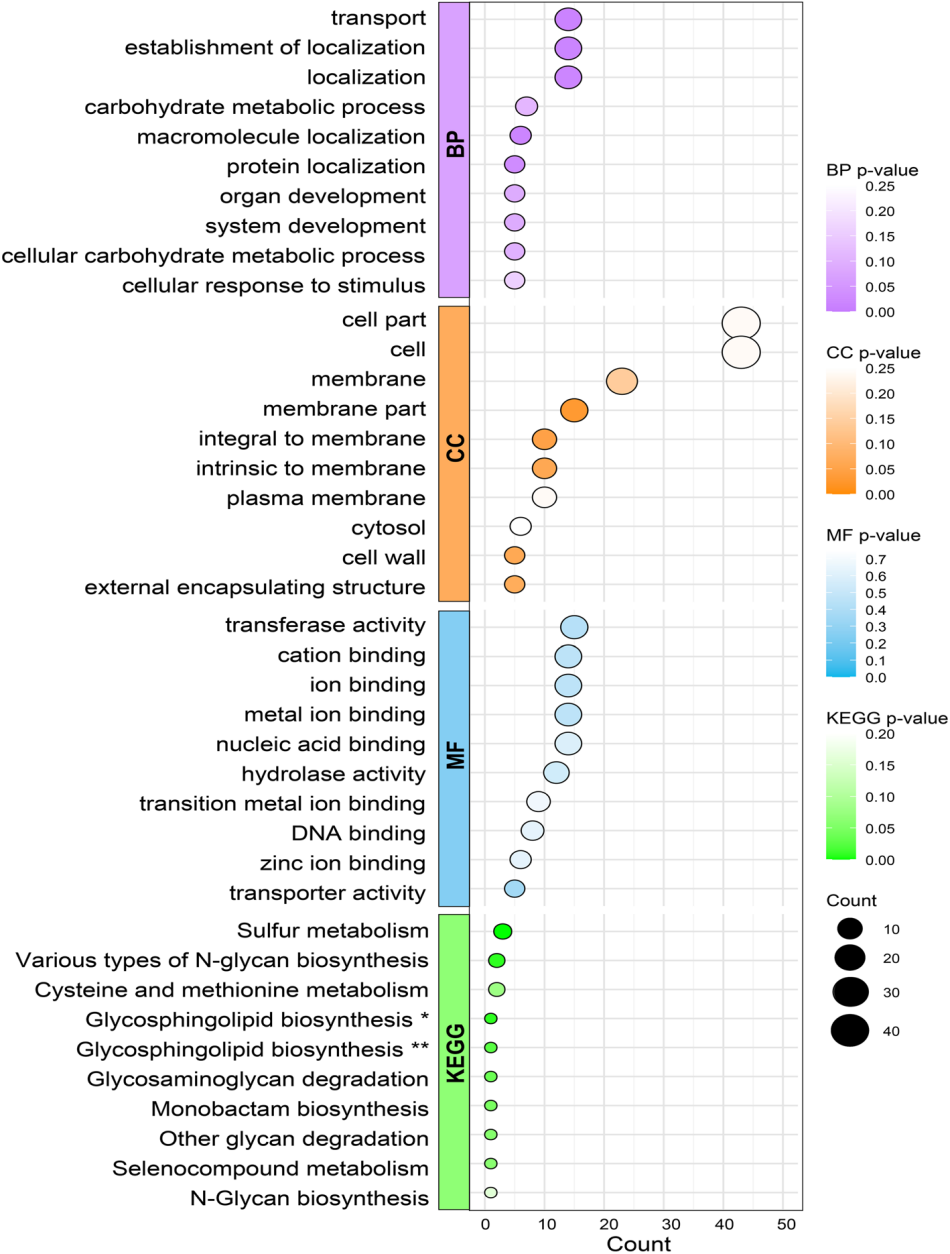
GO and KEGG enrichment analysis of candidate genes. Enrichment results of candidate genes in Gene Ontology (GO) terms (biological process, BP; cellular component, CC; molecular function, MF) and KEGG pathways are shown. The x-axis indicates the number of enriched genes, and the y-axis lists the GO terms or KEGG pathways. Dot size corresponds to the gene count, and color intensity represents the enrichment significance level (-log₁₀(P-value)). KEGG terms marked with an asterisk (*) correspond to “Ganglioside biosynthesis - ganglio series”, and those marked with two asterisks (**) correspond to “Ganglioside biosynthesis - globo and isoglobo series”

Genome-wide association analysis identified 14 significant SNPs associated with maize LA. Within ± 200 kb regions of these SNPs, 342 potential candidate genes were identified, including 134 with functional annotations (Table S2). GO and KEGG enrichment analyses were performed to elucidate the biological functions of these candidates. A total of 83 annotated genes were enriched in 110 GO terms, including “membrane part,” “integral component of membrane,” and “transport” (Fig. 3; Table S3). Additionally, 23 genes were enriched in 28 KEGG pathways, such as sulfur metabolism, glycosphingolipid biosynthesis-ganglio series, and various types of N-glycan biosynthesis (Fig. 3; Table S4). These results provide a basis for the prioritization of candidate genes putatively associated with LA development in maize.

By integrating GO and KEGG enrichment results with maize (MaizeGDB and NCBI) and *Arabidopsis* ortholog annotations, we prioritized 17 high-confidence candidate genes potentially involved in regulating LA from the initial 134 annotated genes (Table S2). Among these, 11 candidate genes located within the confidence intervals of the six stable SNP loci were prioritized. Representative genes including: *GRMZM2G059428* (NAC transcription factor), *GRMZM2G003565* (regulator of chromosome condensation), *AC186329.3_FG009* (tetratricopeptide repeat-like superfamily protein), *GRMZM2G436187* (transducin family protein), *GRMZM2G133173* (ribophorin II), *GRMZM2G133398* (dormancy-associated regulatory protein), *GRMZM2G175983* (auxin efflux carrier), *GRMZM2G036905* (RNA polymerase II elongation complex component), *GRMZM2G092085* (transducin/WD40 repeat protein), *GRMZM2G011156* (cytochrome P450) and *GRMZM2G173630* (Gibberellin-insensitive dwarf protein homolog) (Table 2).

**Fig. 4:**
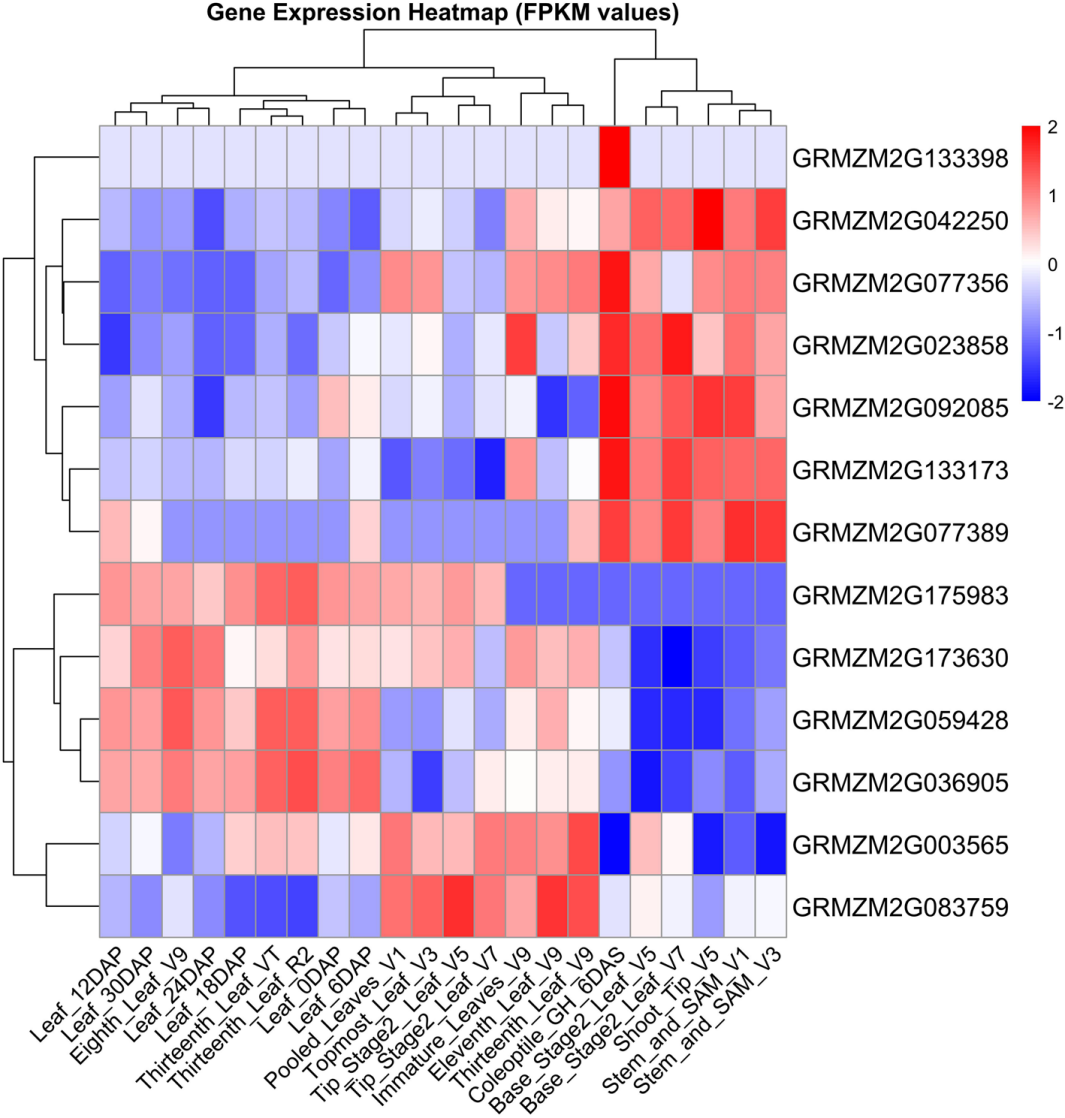
Expression patterns of candidate genes in different tissues of maize B73. Heatmap showing the expression levels of candidate genes across various developmental stages and tissues in the maize reference inbred line B73. The x-axis represents tissue types, and the y-axis lists candidate genes. The color gradient (blue to red) corresponds to expression levels from low to high

To further assess the association of these 17 candidate genes, identified through enrichment analyses and homology-based inference, with LA variation, we analyzed their spatiotemporal expression patterns using publicly available RNA-seq data (Table S5). The results revealed distinct expression profiles across multiple tissues—including coleoptiles, leaf blades, leaf tips, leaf bases, and shoot apical meristems—at various developmental stages (Fig. 4; Table S5). For instance, *GRMZM2G133398* showed markedly higher expression in the coleoptile compared to other tissues. Six genes (*GRMZM2G042250*, *GRMZM2G077356*, *GRMZM2G023858*, *GRMZM2G092085*, *GRMZM2G133173*, and *GRMZM2G077389*) were highly expressed in coleoptiles, leaf bases, stem tips, and shoot apical meristems, suggesting roles in leaf growth and development. In contrast, *GRMZM2G175983*, *GRMZM2G173630*, *GRMZM2G059428*, *GRMZM2G036905*, *GRMZM2G003565*, and *GRMZM2G083759* were more highly expressed in leaves and leaf tips (Fig. 4). No expression was detected for *AC186329.3_FG009* or *GRMZM2G436187* in any profiled tissues, while *GRMZM2G011156* and *GRMZM2G151205* were not expressed in the tissues included in Fig. 4.

### Protein–protein interaction network analysis of candidate genes

**Fig. 5:**
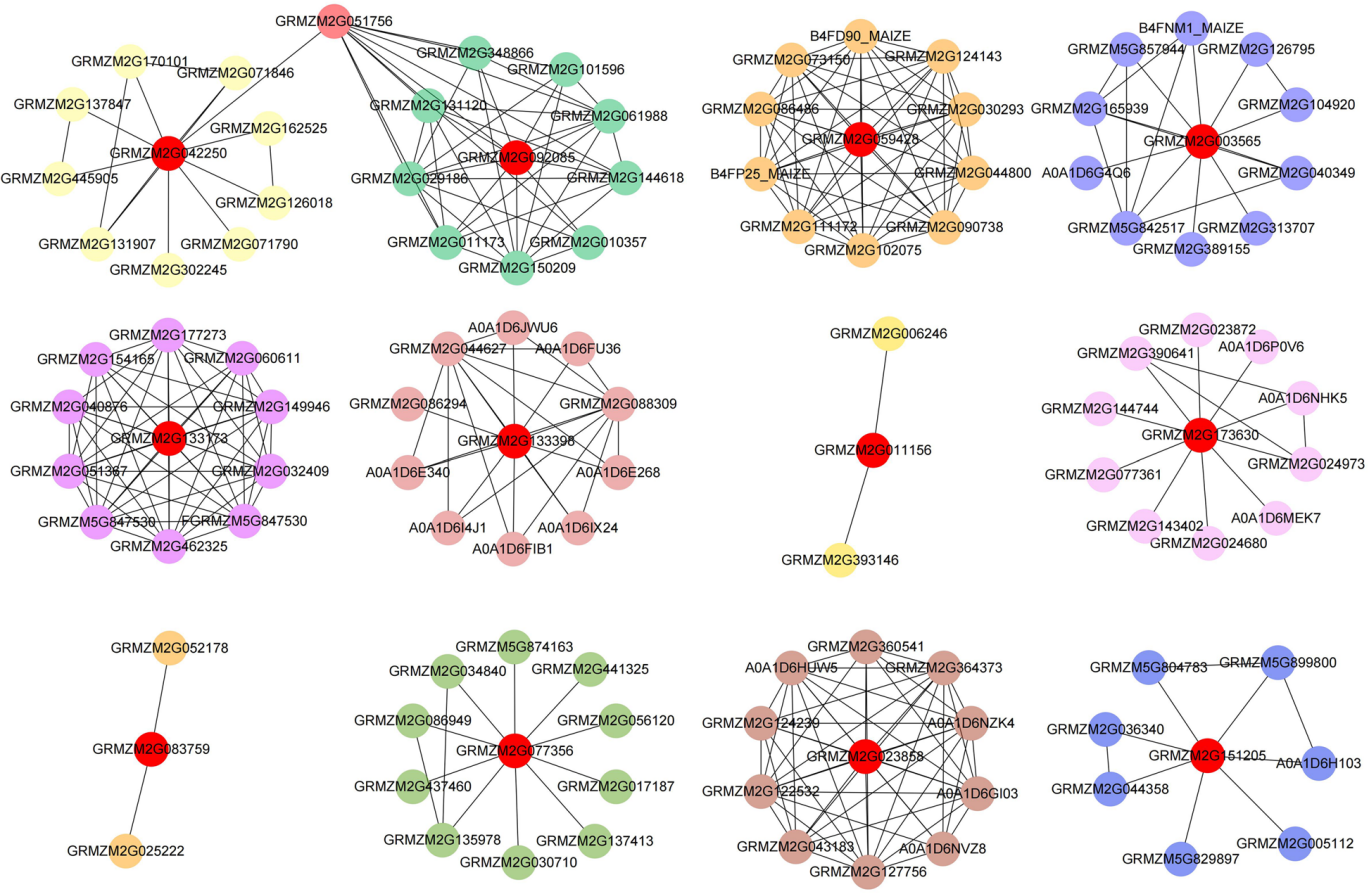
Protein–protein interaction network of candidate gene-encoded proteins. Nodes represent proteins, with those encoded by candidate genes highlighted in red. Black edges indicate known or predicted functional interactions between proteins

To provide a broader context for functional inference by examining interactions between proteins encoded by the candidate genes and other proteins, we constructed a protein–protein interaction (PPI) network using the STRING database (v12.0) with a medium-confidence interaction score (> 0.4) and a maximum of 10 interactors per node to ensure interpretability. Protein sequences for *AC186329.3_FG009* and *GRMZM2G436187* were unavailable in MaizeGDB, while *GRMZM2G175983*, *GRMZM2G036905*, and *GRMZM2G077389* showed limited homology to annotated proteins in STRING, preventing reliable interaction predictions for these five genes. The resulting PPI network indicated that 12 candidate proteins function as network hubs (Fig. 5), forming distinct functional modules potentially involved in key regulatory processes during maize growth and development. Notably, RLD2 (encoded by *GRMZM2G042250*) and A0A1D6Q981 (encoded by *GRMZM2G092085*) both interacted with DCP2 (encoded by *GRMZM2G051756*), an mRNA decapping enzyme whose *Arabidopsis* ortholog mutants exhibit defective venation, impaired leaf development, and premature abscission [46, 47]. Proteins encoded by *GRMZM2G059428* (NAC transcription factor), *GRMZM2G133173* (ribophorin II), and *GRMZM2G023858* formed a highly interconnected hub with multiple partners, suggesting roles in coordinating complex developmental processes. In contrast, *GRMZM2G011156* and *GRMZM2G083759* showed minimal connectivity, each interacting with only two partners, which may reflect specialized functions in specific metabolic pathways (Fig. 5). These differential interaction patterns highlight varying levels of functional integration among candidate genes within the maize developmental regulatory network.

### Analysis of allelic variation effects on LA traits

**Fig. 6:**
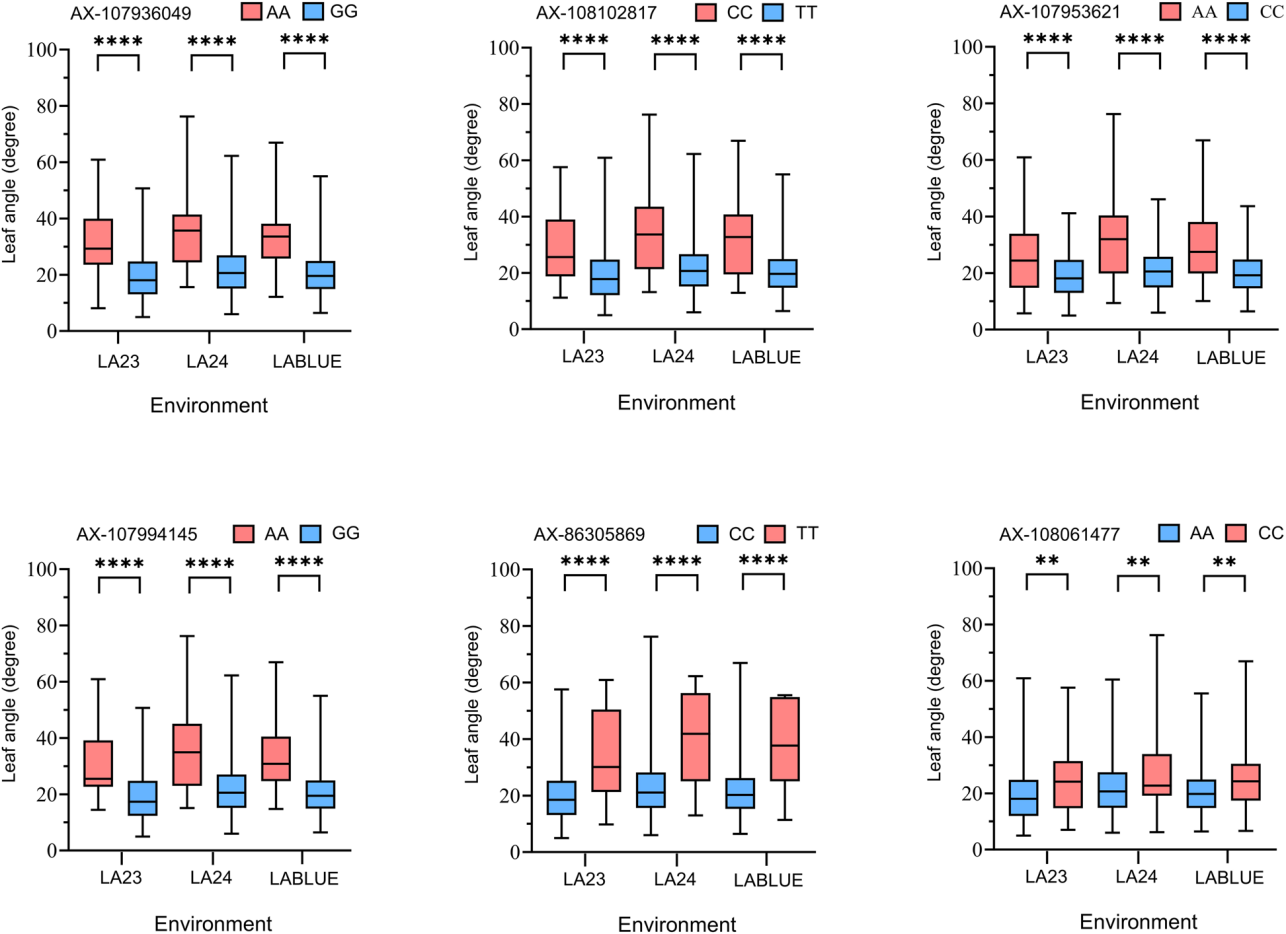
Phenotypic effect of SNP genotypes on leaf angle. Box plots show the distribution of leaf angle values across genotype classes. Blue boxes represent the superior (favorable) genotype at the locus, while red boxes represent the non-superior (unfavorable) genotype. Significance levels: *P < 0.05, **P < 0.01, ****P < 0.0001

To evaluate the functional effects of allelic variation on LA regulation, we conducted a comprehensive analysis of allelic effects at stable SNP loci. As shown in Fig. 6, allelic variations at all six SNP loci showed highly significant associations (*P* < 0.01) with LA phenotypes in three independent environments: LA23, LA24, and LABLUE. At locus AX-107,936,049, inbred lines carrying the G/G allele showed average LA reductions of 13.00°, 13.83°, and 13.23° across the two environments and BLUE compared to those with the A/A allele, indicating that G/G represents a favorable allele. Similarly, for AX-108,102,817, the T/T allele reduced average LA by 10.09°–12.55° relative to the C/C allele. At AX-107,953,621, the C/C allele decreased average LA by 7.39°, 11.87°, and 9.75° compared to the A/A allele. For AX-107,994,145, the G/G allele reduced average LA by 11.97°–13.76° relative to the A/A allele. At AX-86,305,869, the T/T allele resulted in reductions of 15.01°–18.18° compared to the C/C allele. Finally, at AX-108,061,477, the C/C allele reduced LA by 4.78°–5.06° relative to the A/A allele (Fig. 6). These consistent reductions across environments confirm that the identified alleles represent favorable genetic variants for LA improvement.

**Fig. 7:**
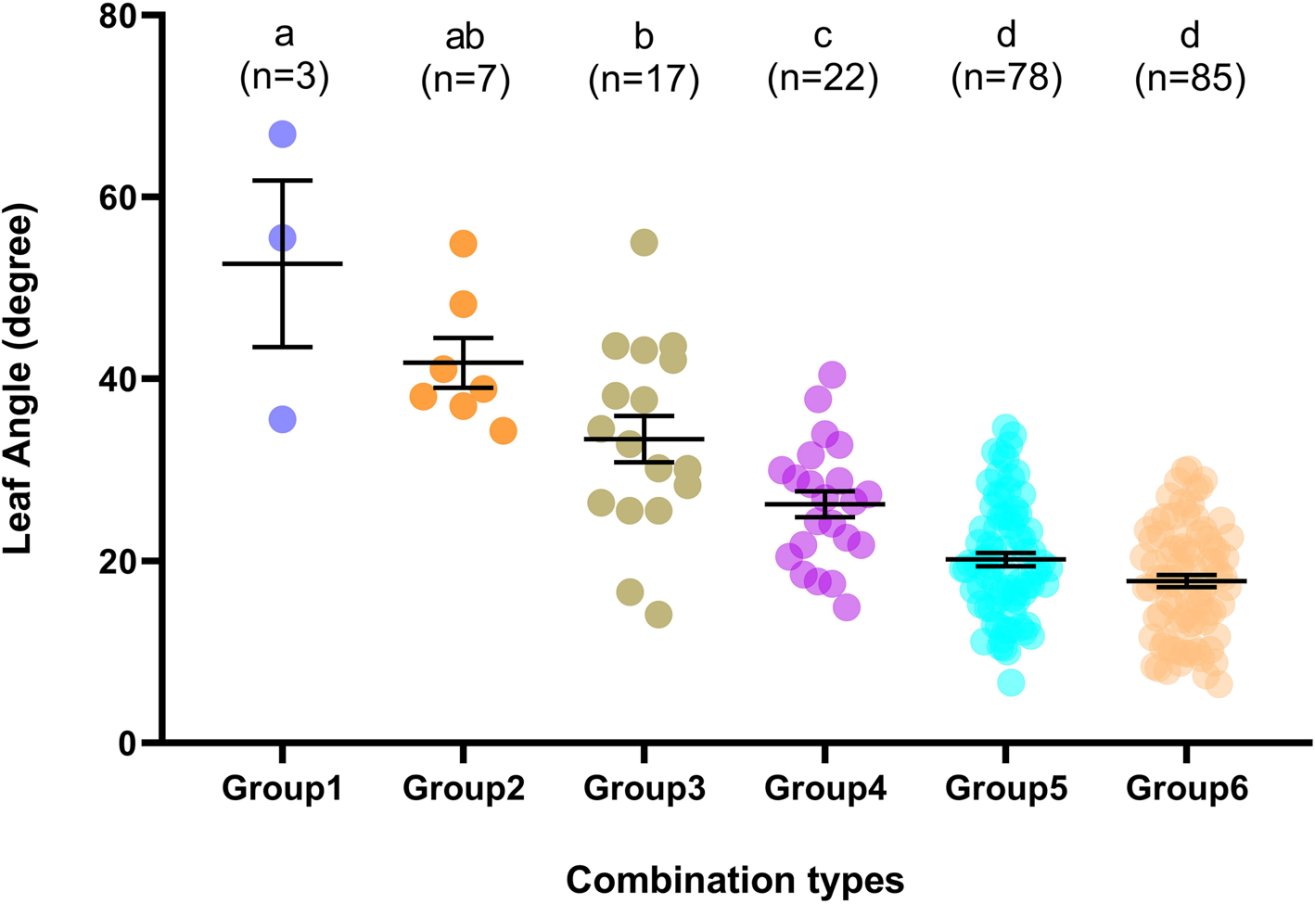
Phenotypic analysis of leaf angle based on the accumulation of favorable alleles at six stable SNP loci. The 212 maize inbred lines were categorized into six groups according to the number of favorable alleles (ranging from 1 to 6) carried at the six stable SNP loci. Leaf angle phenotypes (BLUE values) are compared among the groups. Groups sharing the same letter are not significantly different (Tukey’s test, *P* > 0.05); numbers in parentheses indicate the sample size (number of materials) per group

In this study, the distribution of the six stable SNP loci was further analyzed using 212 inbred lines. The materials were classified into six categories based on the number of favorable alleles carried, ranging from one to six. The corresponding LA phenotypes for the six groups were 52.66 ± 9.17°, 41.77 ± 2.73°, 33.38 ± 2.54°, 26.24 ± 1.42°, 20.17 ± 0.72°, and 17.82 ± 0.66°, respectively (Fig. 7). Notably, materials carrying all six favorable alleles exhibited the smallest LA. Therefore, these six stable SNP loci can be effectively utilized for rapid screening of germplasm with minimal LA, demonstrating promising application potential in maize breeding. It should be noted that Group 1 (carrying 1 favorable alleles) included only three individuals, resulting in a limited sample size. Consequently, the statistical robustness of this observation is constrained, and further validation in larger, independent populations is required (Fig. 7).

### Development and application of KASP markers

**Fig. 8:**
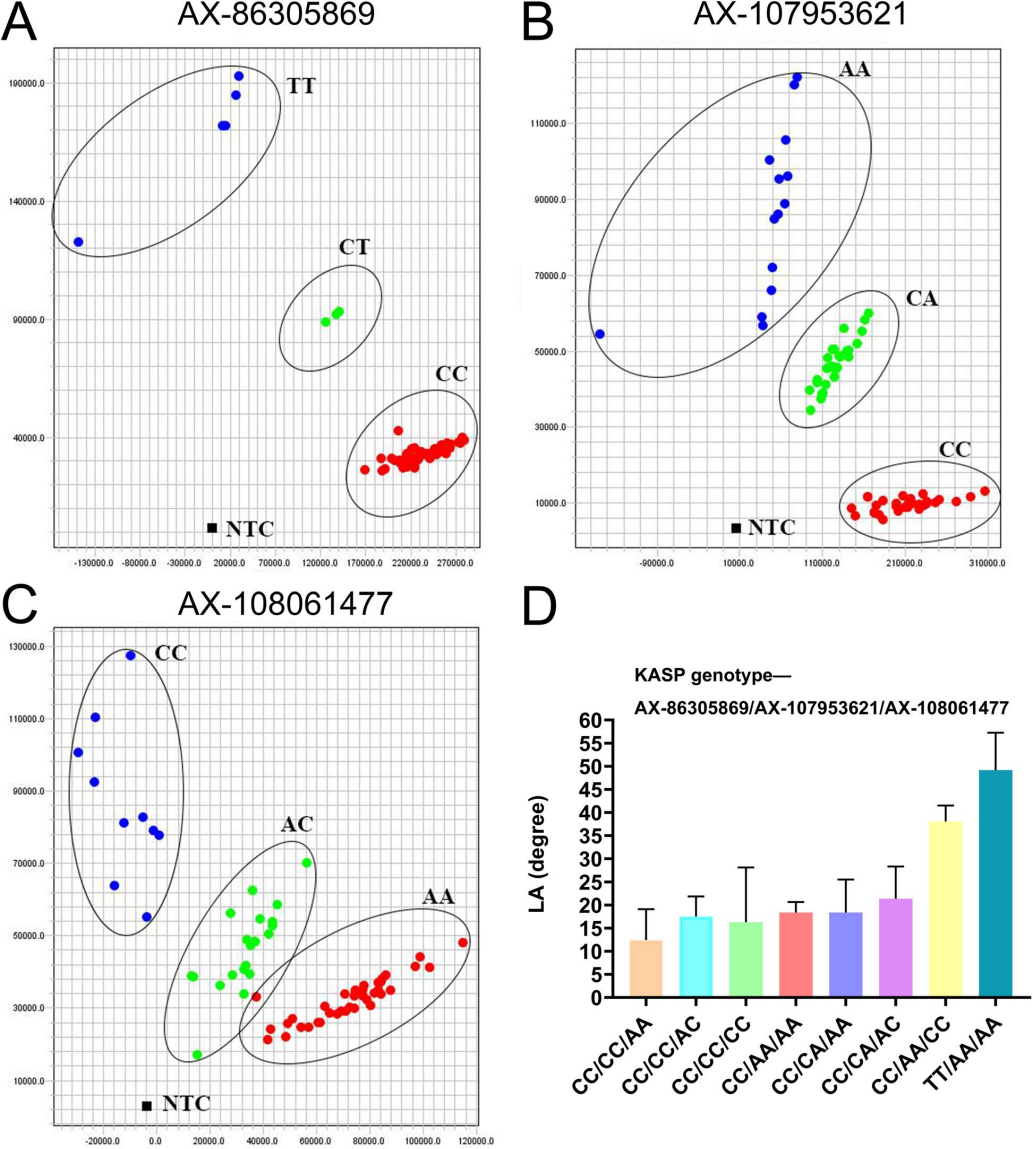
Validation and combined analysis of three KASP markers. **A-C** Genotyping results of KASP markers converted from three stable SNP loci (AX-86305869, AX-107953621, and AX-108061477). **D** Comparison of leaf angle phenotypes among haplotype groups defined by allele combinations at the three loci

Based on the six stable SNP loci, this study designed corresponding KASP primers. Effective KASP markers were successfully developed for the stable loci AX-86,305,869, AX-107,953,621, and AX-108,061,477 located on chromosomes 3, 5, and 6, respectively (Fig. 8A-C; Table S6). Using these markers, genotyping was performed on genomic DNA from 52 maize varieties/lines (including Zhengdan 958, MY73, Anke 985, etc.) and 20 inbred lines (Table S7). The three KASP primer pairs successfully genotyped all tested materials, and the resulting genotypes for the 20 inbred lines were highly consistent with those obtained from the SNP array. Further analysis of variance confirmed significant differences in LA phenotypes among genotype groups defined by these three KASP markers (Fig. 8A-C, Table S7).

Furthermore, combined analysis using these three KASP markers classified all 72 materials into 13 distinct haplotypes, five of which were unique types represented by only a single material (Table S7). Notably, 13 germplasm accessions carrying the haplotype combination CC/CC/AA (corresponding to loci AX-86305869, AX-107953621, and AX-108061477, respectively) exhibited the smallest mean LA (12.46 ± 1.86°) (Fig. 8D; Table S7). The allele types within this combination were confirmed by prior allelic effect analysis to be superior variations favoring reduced LA.

In summary, the stable SNP loci identified on chromosomes 3, 5, and 6 in this study represent superior allelic variations that can significantly reduce LA in maize. Their corresponding KASP markers provide an effective tool for marker-assisted selection in breeding density-tolerant maize varieties.

## Discussion

### Phenotypic analysis of maize LA

In high-yield breeding, increasing planting density to enhance population yield generally holds greater potential than solely improving individual plant productivity [7, 9]. Therefore, developing ideal plant architecture adapted to high-density planting is a key direction in current maize breeding. Studies have shown that a smaller LA promotes more upright leaves, which reduces mutual shading within the canopy, improves light interception efficiency, and enhances ventilation, light penetration, and lodging resistance [7, 9]. Thus, elucidating the genetic basis of LA is of significant importance for ideal plant architecture breeding.

In this study, the LA of three consecutive leaves above the ear was phenotypically evaluated in 212 maize inbred lines. The results showed that this trait was significantly and positively correlated between the two growing seasons, indicating good consistency across environments (Fig. 1). The phenotypic distribution showed a slight high-value tail, indicating limited deviation from normality (Fig. 1); however, mixed linear models are robust to such deviations, and the data were considered suitable for GWAS [48, 49]. The broad-sense heritability of leaf angle was above 80%, aligning with previous reports [50], further confirming that this trait is predominantly controlled by genetic factors and is suitable for genetic dissection and marker-assisted breeding.

### Comparative analysis of GWAS mapping results

Different GWAS models may vary in their power to detect association signals due to distinct strategies for correcting population structure, kinship, and linkage disequilibrium [51, 52]. GLM tends to detect more associations but is prone to false positives, whereas MLM provides stricter control at the cost of reduced power. SUPER, FarmCPU, and BLINK achieve a better balance between detection power and false-positive control, with FarmCPU and BLINK generally showing more stable performance [40]. Therefore, in this study, five models were applied for cross-validation to improve the reliability of GWAS results. In this study, association analysis using 212 maize inbred lines identified 14 SNPs significantly associated with LA. Among them, five SNPs were consistently detected by multiple models. The corresponding Q-Q plots showed no evidence of inflation or over-conservativeness, supporting their classification as stable loci. (Table 2; Table S2), located on chromosomes 1, 2, 3, 4, and 5 respectively. AX-108,061,477 and AX-108,014,790 co-localized within a ± 200 kb interval on chromosome 6 (Table 2; Table S2). Accordingly, AX-108,061,477 was also classified as a stable SNP locus in this study. The fact that these loci were consistently detected across multiple models, indicating a robust association with the LA trait and providing mutual support across different analytical approaches.

Both QTL mapping and GWAS are mainstream methods for dissecting the genetic architecture of complex traits. They both rely on statistical inference of phenotype-genotype associations to locate relevant loci, and their results can be mutually validated to enhance reliability [9, 53, 54]. Among the six stable SNPs identified here, four represent novel loci, while the remaining two show genomic colocalization or proximity (physical distance < 2 Mb) to previously reported QTLs or SNPs. For example, AX-86,305,869 on chromosome 3, detected concurrently by three models, is located at 162,842,787 bp, within 2 Mb of the SNP loci PZE03164583838 and PZE03164630089 reported by Tian et al. [9]. Furthermore, a recent study by Yao et al. reported a leaf-angle-associated locus on chromosome 3 at 163,726,634 bp, which is 883,847 bp away from AX-86,305,869 (i.e., < 1 Mb) [32]. AX-107,994,145 on chromosome 4 (163,227,890 bp), co-detected by the GLM and FarmCPU models, falls within the QTL interval *qLA04-02* (135.34–181.26 Mb) reported by Zhang and Huang [55]. The consistency of these two loci with prior studies further strengthens the credibility of their associations and provides a theoretical basis for molecular marker breeding.

The remaining four stable SNPs, although not directly reported in existing studies, showed robust association signals across multiple models, and candidate genes related to leaf development were identified within their respective ± 200 kb regions (Table S2). Such discrepancies may stem from differences in the genetic materials, environmental conditions, or analytical pipelines employed [56]. Nonetheless, these loci remain valuable references, offering new leads for subsequent functional validation and breeding applications (Table S2).

### Functional analysis of candidate genes and their physiological roles in hormone-mediated leaf development

The regulation of maize LA may involve multiple phytohormonal signaling pathways that potentially regulate cell division, expansion, and differentiation within the leaf collar and midrib regions [7, 24, 57]. From the 134 functionally annotated candidate genes in this study, 17 key genes potentially regulating LA were further screened, 11 of which are located within the ± 200 kb confidence intervals of the six stable SNP loci. These genes are likely involved in regulatory processes integrating auxin (IAA), brassinosteroid (BR), gibberellin (GA), and abscisic acid (ABA) signaling, and may contribute to the modulation of leaf architecture [30, 58].

The gene *GRMZM2G059428*, located within the confidence interval of locus AX-107,936,049, encodes an NAC family transcription factor. Its *Arabidopsis* homolog *ANAC036* is highly expressed in leaves, and its overexpression leads to shortened leaf blades and petioles [59]. NAC transcription factors have been increasingly recognized as integrators of hormonal signals during leaf morphogenesis [58]. These observations suggest that *GRMZM2G059428* may be associated with LA, potentially through effects on adaxial–abaxial development or cell elongation.

The AX-108,102,817 locus harbors several hormonally related candidate genes. *GRMZM2G003565* may encode a regulator of chromosome condensation and could be a direct ortholog of the *Arabidopsis* UV-B receptor *UVR8*. UV-B perception via *UVR8* triggers signaling that can result in shortened petioles and reduced leaf area, leading to a compact plant architecture [60, 61]. Notably, Yang et al. pinpointed the locus *arl1*, which affects maize leaf abaxial rolling, to *Zm00001eb082500* (i.e., *GRMZM2G003565*) within this region, further suggesting a possible role in leaf morphogenesis [62]. *AC186329.3_FG009*, located in the same region, is a homolog of the *Arabidopsis* gene *TPR2. TPR2* has been reported to be involved in axillary and leaf cell development through repression of the branching suppressor *BRANCHED1* [63]. The *Arabidopsis* homolog of *GRMZM2G436187* is *TPL*, which interacts with BR signaling transcription factors *BES1*/*BZR1*, inhibiting the expression of the boundary gene *CUC3*, thereby potentially affecting organ boundary formation [64]. Concurrently, *TPL*-related proteins have also been implicated in the IAA signaling pathway [65], pointing to a possible link between this locus and hormone crosstalk.

At locus AX-86,305,869 on chromosome 3, two candidate genes are potentially related to hormone-associated processes. The *Arabidopsis* ortholog of *GRMZM2G133173*, *HAP6*, encodes a ribophorin implicated in ABA signaling [66], while *GRMZM2G133398* encodes a transcription regulator that simultaneously enhances ABA signaling and negatively regulates GA signaling [67, 68]. These observations indicate that this locus may be associated with hormone-mediated regulation of LA variation, with the balance between GA and ABA signaling potentially contributing to this process, consistent with established mechanisms controlling organ growth [58].

At locus AX-107,994,145, the candidate gene *GRMZM2G175983* encodes a membrane transporter potentially involved in intracellular IAA homeostasis. Its *Arabidopsis* homolog *PIN5* influences leaf vein patterning and cell wall deposition by regulating auxin distribution [69] and interacts with GA signaling to regulate vascular development [70]. This highlights the role of auxin transport in establishing vascular architecture that may contribute to the mechanical support of LA. *GRMZM2G036905* in the same region encodes a component of the RNA polymerase II elongation complex. Mutants of its *Arabidopsis* homolog *ELF1* exhibit increased leaf erectness under specific genetic backgrounds [71], implying a potential role in coordinating transcriptional elongation with leaf inclination regulation. Another gene, *GRMZM2G092085*, is involved in mRNA decapping. Mutants of its *Arabidopsis* homolog *VCS* display leaf developmental defects and abnormal venation patterns [72], supporting its possible involvement in the post-transcriptional regulation of leaf morphogenesis.

The candidate gene *GRMZM2G011156* at locus AX-107,953,621 on chromosome 5 encodes a cytochrome P450 protein. Its *Arabidopsis* homolog participates in the metabolism of IAA biosynthetic precursors [73, 74], which may link it to the auxin biosynthesis pathway, a key regulator of leaf growth and orientation.

The two SNPs on chromosome 6, AX-108,061,477 and AX-108,014,790, are physically adjacent (96.386 kb apart). Given their tight linkage, this study combined them for analysis as a single genetic locus. The candidate gene *GRMZM2G173630* within this interval encodes a gibberellin receptor protein (belonging to the *GID1C* family) with high-affinity binding activity for bioactive *GAs* (e.g., *GA4*). Transcriptome evidence suggests that its expression responds to drought stress, accompanied by changes in DELLA-related genes [75]. In *Arabidopsis*, the mechanism of *GID1* involves, upon *GA* binding, mediating the degradation of DELLA repressor proteins via the 26 S proteasome pathway. This relieves DELLA’s transcriptional repression of downstream growth-related genes, ultimately affecting plant development by regulating processes like cell elongation [76]. Given the role of GA in regulating cell expansion, this pathway may be indirectly associated with variation in LA, although the specific function of *GRMZM2G173630* in maize requires further validation.

Nevertheless, it should be noted that the predicted functions of these genes are primarily inferred from their orthologs and annotations in MaizeGDB and NCBI. As such, their precise biological roles in maize remain unconfirmed, and the mechanisms by which they influence LA are not fully understood. Further experimental validation will be necessary to substantiate these predictions and elucidate their contributions to this trait.

### Breeding implications and limitations of KASP marker development

Several considerations should be noted regarding the breeding application of the identified markers. First, the stable SNPs were detected in a specific set of southeastern China breeding lines; their effects may vary in other genetic backgrounds or environments, and validation in broader germplasm is recommended. Second, some previously reported QTL were not detected in this study, possibly due to fixation of those loci in our panel or differences in linkage disequilibrium patterns. Third, while all six stable SNPs are potentially useful, we attempted to develop KASP markers for all six loci. However, only three loci located on chromosomes 3, 5, and 6 (AX-86305869, AX-107953621, and AX-108061477) successfully yielded KASP markers capable of effectively discriminating among the materials. The overall conversion success rate was 50%, which is lower than the previously reported conversion rate of KASP markers in maize (approximately 71%) [77]. For the unsuccessful loci, the failure can be primarily attributed to the presence of paralogous sequences flanking the target SNPs, leading to a lack of locus specificity in primer design. It is important to note that all six loci were subjected to KASP assay development attempts, rather than a subjective selection of a subset; the final availability of only three markers resulted naturally from technical feasibility screening. Finally, although LA has high heritability (82.7%), marker‑assisted selection offers complementary advantages over phenotypic selection alone, including early‑generation screening, reduced environmental influence, and accelerated line development. Therefore, the validated KASP markers provide practical tools for improving plant architecture in high‑density maize breeding, while the non‑converted SNPs remain targets for future marker optimization or functional validation.

In summary, the candidate genes screened in this study are extensively involved in multiple key biological processes, including the integration of plant hormone signaling (IAA, BR, GA, ABA), transcriptional regulation, cell morphogenesis, and leaf vein development, thereby constituting a complex regulatory network for LA. These results lay a solid theoretical foundation for subsequent gene functional validation and the development of efficient molecular markers.

## Conclusion

In this study, a panel of 212 maize inbred lines was used for GWAS with 58,455 SNP markers through five models (GLM, MLM, SUPER, FarmCPU, and BLINK) implemented in the GAPIT package. A total of 14 SNPs significantly associated with LA were identified, and 342 candidate genes were screened within the 200 kb flanking regions of these SNPs, among which 134 were functionally annotated. Further analysis revealed six stable SNP loci detected by multiple models, including four novel loci reported for the first time. Within the confidence intervals of these six stable loci, 11 functionally annotated candidate genes potentially involved in the variation of LA were identified. For instance, the candidate gene *GRMZM2G036905* has an *Arabidopsis* homolog, *ELF1*, whose double mutant (*elf1-1*/*iws1-1*) exhibits a more upright leaf phenotype; The candidate gene *GRMZM2G003565* has been reported to be related to maize leaf rolling. Additionally, KASP markers were successfully developed for three of the stable SNP loci and validated for their association with LA phenotypes in 52 varieties/lines (including Zhengdan 958, MY73, and Anke 985) and 20 inbred lines. These findings provide new loci and candidate genes for elucidating the genetic basis of LA in maize, laying an important foundation for functional validation of related genes, development of molecular markers, and breeding of density-tolerant maize varieties. It is worth noting that, although stable SNPs associated with LA in maize were identified and corresponding candidate genes were prioritized in this study, the findings are constrained by the specific breeding population analyzed. To fully elucidate the genetic architecture underlying LA, it will be necessary to conduct comparative analyses across diverse germplasm resources, including elite breeding lines, landraces, and wild relatives, and to pursue more comprehensive and in-depth investigations.

## Supplementary Information

Supplementary Material 1

## Data Availability

Data supporting the findings of this study are available within the manuscript and its Supporting Information files.

## References

[1] Stevens T, Madani K. Future climate impacts on maize farming and food security in malawi. Sci Rep. 2016;6(1):36241. 10.1038/srep36241. 10.1038/srep36241 PMC5099946 27824092

[2] FAO. Food Outlook – Biannual report on global food markets. Rome: FAO; 2025. 10.4060/cd5655en.

[3] OECD, FAO. OECD-FAO Agricultural Outlook 2024–2033. Paris and Rome: OECD Publishing; 2024. 10.1787/8825e64d-zh.

[4] Incognito SJP, Maddonni GÁ, López CG. Genetic control of maize plant architecture traits under contrasting plant densities. Euphytica. 2020;216(2):20. 10.1007/s10681-019-2552-9.

[5] Yan J, Warburton M, Crouch J. Association Mapping for Enhancing Maize (*Zea mays* L.) Genetic Improvement. Crop Science. 2011;51(2):433–49. 10.2135/cropsci2010.04.0233.

[6] Li Q, Lai J, Chen Jian, Li L, Song W, Xin B, et al. Decades’ progress and prospects on maize functional genomics and molecular breeding. Sci China Life Sci. 2025; 68(12): 3509–74. 10.1007/s11427-025-3022-6. 10.1007/s11427-025-3022-6 41111077

[7] Cao Y, Zhong Z, Wang H, Shen R. Leaf angle: a target of genetic improvement in cereal crops tailored for high-density planting. Plant Biotechnology Journal. 2022;20(3):426–36. 10.1111/pbi.13780. 10.1111/pbi.13780 PMC8882799 35075761

[8] Wang Q, Guo Q, Shi Q, Yang H, Liu M, Niu Y, et al. Histological and single-nucleus transcriptome analyses reveal the specialized functions of ligular sclerenchyma cells and key regulators of leaf angle in maize. Molecular Plant. 2024;17(6):920–34. 10.1016/j.molp.2024.05.001. 10.1016/j.molp.2024.05.001 38720461

[9] Tian F, Bradbury PJ, Brown PJ, Hung H, Sun Q, Flint-Garcia S, et al. Genome-wide association study of leaf architecture in the maize nested association mapping population. Nat Genet. 2011;43(2):159–62. 10.1038/ng.746. 10.1038/ng.746 21217756

[10] 10. Bai Y, Yang Y, Zhu Y, Li Hongjie, Xue J, Zhang Renhe. Effect of planting density on light interception within canopy and grain yield of different plant types of maize. Acta Agronomica Sinica. 2019;45(12):1868–79. 10.3724/SP.J.1006.2019.93011.

[11] 11. Chen H, Gong X, Guo Y, Yu J, Li W-X, Du Q. *ZmbZIP27* regulates nitrogen-mediated leaf angle by modulating lignin deposition in maize. The Crop Journal. 2024;12(5):1404–13. 10.1016/j.cj.2024.09.004.

[12] 12. Zhao J, Sun. Re-thinking on Breeding Objective and Technica l Route of Super Maize. Journal of Maize Sciences. 2007;28:21–3.

[13] 13. Zhang X, Dong R, Xu Y, Du X, Ma L. Genome‑wide association study identifies GhF3’H affects the spiraeoside biosynthesis in waste cotton (Gossypium hirsutum L.) flowers. Industrial Crops and Products. 2023;204:117330. 10.1016/j.indcrop.2023.117330.

[14] 14. Zhang J, Gu R, Miao X, Schmidt RH, Xu Z, Lu J, et al. GWAS-based population genetic analysis identifies bZIP29 as a heterotic gene in maize. Plant Communications. 2025;6(5):101289. 10.1016/j.xplc.2025.101289. 10.1016/j.xplc.2025.101289 PMC12143148 39985171

[15] 15. Raman H, Bai Z, McVittie B, Mukherjee S, Goold HD, Zhang Y, et al. Genome-Wide Association Study Elucidates the Genetic Architecture of Manganese Tolerance in *Brassica napus*. Plant Cell & Environment. 2025;:pce.15433. 10.1111/pce.15433. 10.1111/pce.15433 39948049

[16] 16. Sun H, Yao Q, Hai M, Li T, Sun J, Liu Z, et al. Elite Alleles of EPE1 Identified via Genome-wide Association Studies Increase Panicle Elongation Length in Rice. Rice. 2025;18(1):4. 10.1186/s12284-025-00759-7. 10.1186/s12284-025-00759-7 PMC11780047 39878928

[17] 17. Liu D, Lu S, Tian R, Zhang X, Dong Q, Ren H, et al. Mining genomic regions associated with stomatal traits and their candidate genes in bread wheat through genome-wide association study (GWAS). Theor Appl Genet. 2025;138(1):20. 10.1007/s00122-024-04814-7. 10.1007/s00122-024-04814-7 39774685

[18] 18. Korte A, Farlow A. The advantages and limitations of trait analysis with GWAS: a review. Plant Methods. 2013;9(1):29. 10.1186/1746-4811-9-29. 10.1186/1746-4811-9-29 PMC3750305 23876160

[19] Lu S, Zhang M, Zhang Z, Wang Z, Wu N, Song Y, et al. Screening and verification of genes associated with leaf angle and leaf orientation value in inbred maize lines. Plos One. 2018;13(12):e0208386. 10.1371/journal.pone.0208386. 30532152 10.1371/journal.pone.0208386 PMC6285979

[20] Duan H, Li J, Sun Y, Xiong X, Sun L, Li W, et al. Candidate loci for leaf angle in maize revealed by a combination of genome-wide association study and meta-analysis. Front Genet. 2022;13:1004211. 10.3389/fgene.2022.1004211. 36437932 10.3389/fgene.2022.1004211 PMC9691904

[21] Peng B, Zhao X, Wang Y, Li C, Li Y, Zhang D, et al. Genome-wide association studies of leaf angle in maize. Mol Breed. 2021;41(8):50. 10.1007/s11032-021-01241-0. 37309541 10.1007/s11032-021-01241-0 PMC10236034

[22] Brant EJ, Baloglu MC, Parikh A, Altpeter F. CRISPR/Cas9 mediated targeted mutagenesis of *LIGULELESS-1* in sorghum provides a rapidly scorable phenotype by altering leaf inclination angle. Biotechnol J. 2021;16(11):2100237. 10.1002/biot.202100237. 10.1002/biot.202100237 34343415

[23] Harper L, Freeling M. Interactions of liguleless1 and liguleless2 function during ligule induction in maize. Genetics. 1996;144(4):1871–82. 10.1093/genetics/144.4.1871. 8978070 10.1093/genetics/144.4.1871 PMC1207734

[24] Wang X, Wang X, Sun S, Tu X, Lin K, Qin L, et al. Characterization of regulatory modules controlling leaf angle in maize. Plant Physiol. 2022;190(1):500–15. 10.1093/plphys/kiac308. 35758633 10.1093/plphys/kiac308 PMC9434308

[25] Zheng B, Zhou B, Wu G, Xu Y. Advance in the study of leaf adaxial-abaxial polarity development in plants. Plant Physiology Journal. 2015;51(12):2091–100. 10.13592/j.cnki.ppj.2015.0443.

[26] Shi Q, Xia Y, Wang Q, Lv K, Yang H, Cui L, et al. Phytochrome B interacts with LIGULELESS1 to control plant architecture and density tolerance in maize. Mol Plant. 2024;17(8):1255–71. 10.1016/j.molp.2024.06.014. 38946140 10.1016/j.molp.2024.06.014

[27] Jafari F, Wang B, Wang H, Zou J. Breeding maize of ideal plant architecture for high-density planting tolerance through modulating shade avoidance response and beyond. JIPB. 2024;66(5):849–64. 10.1111/jipb.13603. 38131117 10.1111/jipb.13603

[28] Tian J, Wang C, Xia J, Wu L, Xu G, Wu W, et al. Teosinte ligule allele narrows plant architecture and enhances high-density maize yields. Science. 2019;365(6454):658–64. 10.1126/science.aax5482. 31416957 10.1126/science.aax5482

[29] Wei L, Zhang X, Zhang Z, Liu H, Lin Z. A new allele of the *Brachytic2* gene in maize can efficiently modify plant architecture. Heredity. 2018;121(1):75–86. 10.1038/s41437-018-0056-3. 29472693 10.1038/s41437-018-0056-3 PMC5997708

[30] Ren Z, Wu L, Ku L, Wang H, Zeng H, Su H, et al. ZmILI1 regulates leaf angle by directly affecting liguleless1 expression in maize. Plant Biotechnol J. 2020;18(4):881–3. 10.1111/pbi.13255. 31529573 10.1111/pbi.13255 PMC7061864

[31] Strable J, Wallace JG, Unger-Wallace E, Briggs S, Bradbury PJ, Buckler ES, et al. Maize YABBY genes drooping leaf1 and drooping leaf2 regulate plant architecture. Plant Cell. 2017;29(7):1622–41. 10.1105/tpc.16.00477. 28698237 10.1105/tpc.16.00477 PMC5559738

[32] Yao W, Ku L, Wang B, Ren Z, Su H, Li C, et al. Breeding ideotype maize with enhanced yield through genomics-guided pyramiding of favorable alleles. Nat Genet. 2026;58(4):916–27. 10.1038/s41588-026-02522-0. 41845062 10.1038/s41588-026-02522-0

[33] Mohammadi M, Xavier A, Beckett T, Beyer S, Chen L, Chikssa H, et al. Identification, deployment, and transferability of quantitative trait loci from genome-wide association studies in plants. Current Plant Biology. 2020;24:100145. 10.1016/j.cpb.2020.100145.

[34] Pang Y, Liu C, Wang D, St. Amand P, Bernardo A, Li W, et al. High-resolution genome-wide association study identifies genomic regions and candidate genes for important agronomic traits in wheat. Mol Plant. 2020;13(9):1311–27. 10.1016/j.molp.2020.07.008. 32702458 10.1016/j.molp.2020.07.008

[35] Wang C, He W, Li K, Yu Y, Zhang X, Yang S, et al. Genetic diversity analysis and GWAS of plant height and ear height in maize inbred lines from South-East China. Plants. 2025;14(3):481. 10.3390/plants14030481. 39943042 10.3390/plants14030481 PMC11820090

[36] Merk HL, Yarnes SC, Van Deynze A, Tong N, Menda N, Mueller LA, et al. Trait diversity and potential for selection indices based on variation among regionally adapted processing tomato germplasm. J Amer Soc Hort Sci. 2012;137(6):427–37. 10.21273/jashs.137.6.427.

[37] Chen L, He W, Yu Y, Wang Y, Zhai X, Ling X, et al. Molecular mapping and candidate gene identification of two major quantitative trait loci associated with silique length in oilseed rape (*Brassica napus* L.). Mol Breed. 2024;44(4):26. 10.1007/s11032-024-01464-x. 38516204 10.1007/s11032-024-01464-x PMC10951173

[38] Tian H, Yang Y, Yi H, Xu L, He H, Fan Y, et al. New resources for genetic studies in maize (*Zea mays* L.): a genome-wide Maize6H‐60K single nucleotide polymorphism array and its application. Plant J. 2021;105(4):1113–22. 10.1111/tpj.15089. 33225500 10.1111/tpj.15089

[39] Huang M, Liu X, Zhou Y, Summers RM, Zhang Z. BLINK: a package for the next level of genome-wide association studies with both individuals and markers in the millions. Gigascience. 2019;8(2):giy154. 10.1093/gigascience/giy154. 30535326 10.1093/gigascience/giy154 PMC6365300

[40] Wang J, Zhang Z. GAPIT version 3: boosting power and accuracy for genomic association and prediction. Genomics Proteomics Bioinformatics. 2021;19(4):629–40. 10.1016/j.gpb.2021.08.005. 34492338 10.1016/j.gpb.2021.08.005 PMC9121400

[41] Yu J, Pressoir G, Briggs WH, Vroh Bi I, Yamasaki M, Doebley JF, et al. A unified mixed-model method for association mapping that accounts for multiple levels of relatedness. Nat Genet. 2006;38(2):203–8. 10.1038/ng1702. 16380716 10.1038/ng1702

[42] Wang Q, Tian F, Pan Y, Buckler ES, Zhang Z. A SUPER powerful method for genome wide association study. PLoS One. 2014;9(9):e107684. 10.1371/journal.pone.0107684. 25247812 10.1371/journal.pone.0107684 PMC4172578

[43] Liu X, Huang M, Fan B, Buckler ES, Zhang Z. Iterative usage of fixed and random effect models for powerful and efficient genome-wide association studies. PLoS Genet. 2016;12(2):e1005767. 10.1371/journal.pgen.1005767. 26828793 10.1371/journal.pgen.1005767 PMC4734661

[44] Shan S, Xu F, Brenig B. Genome-wide association studies reveal neurological genes for dog herding, predation, temperament, and trainability traits. Front Vet Sci. 2021;8:693290. 10.3389/fvets.2021.693290. 34368281 10.3389/fvets.2021.693290 PMC8335642

[45] Yu Y, Rizwan A, Sun T, Wang D, Cui N, Chen L, et al. GWAS-based prediction of genes regulating the weight of mobilized reserved seeds in sweet corn. Agronomy Basel. 2024;14(11):2648. 10.3390/agronomy14112648.

[46] Goeres DC, Van Norman JM, Zhang W, Fauver NA, Spencer ML, Sieburth LE. Components of the *Arabidopsis* mRNA decapping complex are required for early seedling development. Plant Cell. 2007;19(5):1549–64. 10.1105/tpc.106.047621. 17513503 10.1105/tpc.106.047621 PMC1913740

[47] Xu J, Yang J-Y, Niu Q-W, Chua N-H. *Arabidopsis* DCP2, DCP1, and varicose form a decapping complex required for postembryonic development. Plant Cell. 2007;18(12):3386–98. 10.1105/tpc.106.047605. 10.1105/tpc.106.047605 PMC1785416 17158604

[48] Warrington NM, Tilling K, Howe LD, Paternoster L, Pennell CE, Wu YY, et al. Robustness of the linear mixed effects model to error distribution assumptions and the consequences for genome-wide association studies. Stat Appl Genet Mol Biol. 2014;13(5):1–21. 10.1515/sagmb-2013-0066. 25153607 10.1515/sagmb-2013-0066

[49] Schielzeth H, Dingemanse NJ, Nakagawa S, Westneat DF, Allegue H, Teplitsky C, et al. Robustness of linear mixed-effects models to violations of distributional assumptions. Methods Ecol Evol. 2020;11(9):1141–52. 10.1111/2041-210X.13434.

[50] Peng B, Zhao X, Wang Y, Yuan W, Li C, Li Y, et al. Genome-wide association studies of leaf orientation value in maize. Acta Agron Sin. 2020;46(6):819–31.

[51] 51. Liu D, Li X, Wang L, Pei Q, Zhao J, Sun D, et al. Genome-wide association studies of body size traits in tibetan sheep. BMC Genomics. 2024;25(1):739–54. 10.1186/s12864-024-10633-3. 10.1186/s12864-024-10633-3 PMC11290296 39080522

[52] Rehman AU, Iso-Touru T, Junkers J, Rantanen M, Karhu S, Fischer D, et al. Multi-model GWAS reveals key loci for horticultural traits in reconstructed garden strawberry. Physiol Plant. 2024;176(4):e14440. 10.1111/ppl.14440. 39030778 10.1111/ppl.14440

[53] Li C, Song Y, Zhu Y, Cao M, Han X, Fan J, et al. GWAS analysis reveals candidate genes associated with density tolerance (ear leaf structure) in maize (*Zea mays* L.). J Integr Agric. 2025;24(6):2046–62. 10.1016/j.jia.2024.01.023.

[54] Li P, Zhang Z, Xiao G, Zhao Z, He K, Yang X, et al. Genomic basis determining root system architecture in maize. Theor Appl Genet. 2024;137(5):102. 10.1007/s00122-024-04606-z. 38607439 10.1007/s00122-024-04606-z

[55] Zhang N, Huang X. Mapping quantitative trait loci and predicting candidate genes for leaf angle in maize. PLoS One. 2021;16(1):e0245129. 10.1371/journal.pone.0245129. 33406127 10.1371/journal.pone.0245129 PMC7787474

[56] Boakyewaa Adu G, Badu-Apraku B, Akromah R, Garcia-Oliveira AL, Awuku FJ, Gedil M. Genetic diversity and population structure of early-maturing tropical maize inbred lines using SNP markers. PLoS One. 2019;14(4):e0214810. 10.1371/journal.pone.0214810. 30964890 10.1371/journal.pone.0214810 PMC6456193

[57] Zhang Q, Zhang P, Xu C, Cong Y, Liu L. Advances in genetic researches and molecular regulation mechanism of leaf angle in maize. J Agric Sci Technol. 2021;23(10):15–24. 10.13304/j.nykjdb.2021.0121.

[58] Zhao B, Liu Q, Wang B, Yuan F. Roles of phytohormones and their signaling pathways in leaf development and stress responses. J Agric Food Chem. 2021;69(12):3566–84. 10.1021/acs.jafc.0c07908. 33739096 10.1021/acs.jafc.0c07908

[59] Kato H, Motomura T, Komeda Y, Saito T, Kato A. Overexpression of the NAC transcription factor family gene ANAC036 results in a dwarf phenotype in *Arabidopsis thaliana*. J Plant Physiol. 2010;167(7):571–7. 10.1016/j.jplph.2009.11.004. 19962211 10.1016/j.jplph.2009.11.004

[60] Cunningham N, Crestani G, Csepregi K, Coughlan NE, Jansen MAK. Exploring the complexities of plant UV responses; distinct effects of UV-A and UV-B wavelengths on *Arabidopsis* rosette morphology. Photochem Photobiol Sci. 2024;23(7):1251–64. 10.1007/s43630-024-00591-w. 38736023 10.1007/s43630-024-00591-w PMC11224116

[61] Liu W, Giuriani G, Havlikova A, Li D, Lamont DJ, Neugart S, et al. Phosphorylation of *Arabidopsis UVR8* photoreceptor modulates protein interactions and responses to UV-B radiation. Nat Commun. 2024;15(1):1221. 10.1038/s41467-024-45575-7. 38336824 10.1038/s41467-024-45575-7 PMC10858049

[62] Yang M, Huang A, Wen R, Tian S, Mo R, Zhai R, et al. Identification of the arl1 locus controlling leaf rolling and its application in maize breeding. Mol Breed. 2025;45(1):9. 10.1007/s11032-024-01534-0. 39763573 10.1007/s11032-024-01534-0 PMC11700961

[63] Wang L, Wang B, Jiang L, Liu X, Li X, Lu Z, et al. Strigolactone signaling in Arabidopsis regulates shoot development by targeting D53-like SMXL repressor proteins for ubiquitination and degradation. Plant Cell. 2015;27(11):3128–42. 10.1105/tpc.15.00605. 26546446 10.1105/tpc.15.00605 PMC4682305

[64] Espinosa-Ruiz A, Martínez C, De Lucas M, Fàbregas N, Bosch N, Caño-Delgado AI, et al. TOPLESS mediates brassinosteroid control of shoot boundaries and root meristem development in *Arabidopsis thaliana*. Development. 2017;dev.143214. 10.1242/dev.143214. 10.1242/dev.143214 28320734

[65] Cho H-T, Lee M, Choi H-S, Maeng K-H, Lee K, Lee H-Y, et al. A dose-dependent bimodal switch by homologous Aux/IAA transcriptional repressors. Mol Plant. 2024;17(9):1407–22. 10.1016/j.molp.2024.07.014. 39095993 10.1016/j.molp.2024.07.014

[66] Giordano L, Schimmerling M, Panabières F, Allasia V, Keller H. The exodomain of the impaired oomycete susceptibility 1 receptor mediates both endoplasmic reticulum stress responses and abscisic acid signalling during downy mildew infection of *Arabidopsis*. Mol Plant Pathol. 2022;23(12):1783–91. 10.1111/mpp.13265. 36103373 10.1111/mpp.13265 PMC9644275

[67] McCarty DR, Hattori T, Carson CB, Vasil V, Lazar M, Vasil IK. The Viviparous-1 developmental gene of maize encodes a novel transcriptional activator. Cell. 1991;66(5):895–905. 10.1016/0092-8674(91)90436-3. 1889090 10.1016/0092-8674(91)90436-3

[68] Xian B, Rehmani MS, Fan Y, Luo X, Zhang R, Xu J, et al. The ABI4-RGL2 module serves as a double agent to mediate the antagonistic crosstalk between ABA and GA signals. New Phytol. 2024;241(6):2464–79. 10.1111/nph.19533. 38287207 10.1111/nph.19533

[69] Verna C, Sawchuk MG, Linh NM, Scarpella E. Control of vein network topology by auxin transport. BMC Biol. 2015;13(1):94. 10.1186/s12915-015-0208-3. 26560462 10.1186/s12915-015-0208-3 PMC4641347

[70] Johnsson C, Jin X, Xue W, Dubreuil C, Lezhneva L, Fischer U. The plant hormone auxin directs timing of xylem development by inhibition of secondary cell wall deposition through repression of secondary wall NAC-domain transcription factors. Physiol Plant. 2019;165(4):673–89. 10.1111/ppl.12766. 29808599 10.1111/ppl.12766 PMC7379297

[71] Markusch H, Michl-Holzinger P, Obermeyer S, Thorbecke C, Bruckmann A, Babl S, et al. Elongation factor 1 is a component of the *Arabidopsis*RNA polymerase II elongation complex and associates with a subset of transcribed genes. New Phytol. 2023;238(1):113–24. 10.1111/nph.18724. 36627730 10.1111/nph.18724

[72] Deyholos MK, Cavaness GF, Hall B, King E, Punwani J, Van Norman J, et al. VARICOSE, a WD-domain protein, is required for leaf blade development. Development. 2003;130(26):6577–88. 10.1242/dev.00909. 14660546 10.1242/dev.00909

[73] Crane RA, Cardénas Valdez M, Castaneda N, Jackson CL, Riley CJ, Mostafa I, et al. Negative regulation of age-related developmental leaf senescence by the IAOx pathway, PEN1, and PEN3. Front Plant Sci. 2019;10:1202. 10.3389/fpls.2019.01202. 31649689 10.3389/fpls.2019.01202 PMC6792297

[74] Zhao Y, Hull AK, Gupta NR, Goss KA, Alonso J, Ecker JR, et al. Trp-dependent auxin biosynthesis in *Arabidopsis*: involvement of cytochrome P450s CYP79B2 and CYP79B3. Genes Dev. 2002;16(23):3100–12. 10.1101/gad.1035402. 12464638 10.1101/gad.1035402 PMC187496

[75] Wang B, Liu C, Zhang D, He C, Zhang J, Li Z. Effects of maize organ-specific drought stress response on yields from transcriptome analysis. BMC Plant Biol. 2019;19(1):335. 10.1186/s12870-019-1941-5. 31370805 10.1186/s12870-019-1941-5 PMC6676540

[76] Griffiths J, Murase K, Rieu I, Zentella R, Zhang Z-L, Powers SJ, et al. Genetic characterization and functional analysis of the GID1 gibberellin receptors in *Arabidopsis*. Plant Cell. 2007;18(12):3399–414. 10.1105/tpc.106.047415. 10.1105/tpc.106.047415 PMC1785415 17194763

[77] Jagtap AB, Vikal Y, Johal GS. Genome-wide development and validation of cost-effective KASP marker assays for genetic dissection of heat stress tolerance in maize. Int J Mol Sci. 2020;21(19):7386. 10.3390/ijms21197386. 33036291 10.3390/ijms21197386 PMC7582619

